# SIRT3 deficiency leads to induction of abnormal glycolysis in diabetic kidney with fibrosis

**DOI:** 10.1038/s41419-018-1057-0

**Published:** 2018-09-24

**Authors:** Swayam Prakash Srivastava, Jinpeng Li, Munehiro Kitada, Hiroki Fujita, Yuichiro Yamada, Julie E. Goodwin, Keizo Kanasaki, Daisuke Koya

**Affiliations:** 1 Department of Diabetology & Endocrinology, Kanazawa Medical University, Uchinada, Ishikawa 920-0293 Japan; 2 Department of Pediatrics (Nephrology) Yale University School of Medicine, New Haven, CT 06520 USA; 3 Division of Anticipatory Molecular Food Science and Technology, Kanazawa Medical University, Uchinada, Ishikawa 920-0293 Japan; 4 Department of Endocrinology, Diabetes and Geriatric Medicine, Akita University Graduate School of Medicine, Akita, 010-8543 Japan

## Abstract

The regulation of aberrant glucose metabolism in diabetes associated-kidney fibrosis is not well known. In this study we found the suppression of SIRT3 protein level in diabetic kidney, displays responsibility in fibrogenic programming associated with aberrant glycolysis and such abnormal glycolysis is the therapeutic target in diabetes associated-kidney fibrosis. When analyzing different strains of streptozotocin-induced diabetic mice model (fibrotic model: CD-1, less fibrotic model: C57Bl6), we found SIRT3 suppression was associated with kidney fibrosis in fibrotic CD-1; further SIRT3 suppression by systemic administration of SIRT3 siRNA in the diabetic mice, showed profound fibrogenic phenotype in the kidney. Such suppression in SIRT3 was associated with the induction of transforming growth factor-β (TGF-β)/smad signaling, higher level of HIF1α accumulation and PKM2 dimer formation; these alterations subsequently led to abnormal glycolysis and linked abnormal mesenchymal transformations in vivo and in vitro. Inhibition of such aberrant glycolysis suppressed fibrogenic programming and restored SIRT3 level as well. Such aberrant glycolysis was confirmed in the KK/Ta-Ins2Akita mouse, the mouse model of progressive diabetic kidney disease. These data demonstrate that SIRT3 deficiency promotes abnormal glycolysis which is responsible for the fibrogenic pathway in diabetic kidney. Restoration of SIRT3 could be an alternative strategy in combating diabetes associated-kidney fibrosis via inhibition of aberrant glycolysis.

## Introduction

Kidney fibrosis is the final common outcome of diabetic kidney disease and leading cause of end stage renal disease worldwide^1^. Kidney fibrosis is characterized by loss of capillary networks, accumulation of collagens, activated myofibroblasts and inflammatory cells^2–4^. Kidney fibroblasts play an important role during the fibrotic process, but the origin of fibroblast is still obscure^4–9^. Researchers have proposed that myofibroblasts formation could be due to activation of resident fibroblasts and activation of mesenchymal transition programs in neighboring cells^4–9^.

SIRT3 is a major mitochondrial deacetylase that targets several diverse enzymes involved in central metabolism resulting in the activation of many oxidative pathways^10^. SIRT3 functions as a tumor suppressor by maintaining genomic stability^11^ and blocks the characteristics of organ fibrosis by regulating TGF-β/smad signaling^12–16^.

The renal tubular epithelial cells require the high level of the baseline energy for its functioning. The proximal tubules reabsorb glucose from the urine and also can synthesize glucose by gluconeogenesis but mostly do not metabolize glucose for its own energy requirements. Although most of its energy is derived from oxidation of free fatty acids. However, during the cellular transformation process, the metabolic switch is altered^17–21^. Therefore the utilization and selection of energy source in these damaged cells is a matter of debate^17–21^. The altered metabolic switch may contribute to the accumulation of myofibroblast precursors and can support the fibroblast survival and proliferation^2,18,22,23^. Clinical data suggests that diabetic kidney is associated with higher lactate level and decrease in redox potential, a shift similar to Warburg metabolism in cancer cells^24^. Induction of glycolysis is associated with reduced mitochondrial number and suppressed mitochondrial physiology in the atrophic tubular cells after ischemic acute kidney injury (AKI) in the Sprague Dawley rats^25^. Some of the alterations of energy metabolism reported so far in the mouse models of AKI have shown increased lactate release into the interstitium after ischemic AKI^26^, elevated pyruvate kinase in the kidney homogenate after ischemia reperfusion injury^27^, diminished fatty acid oxidation after folic acid nephropathy^28^ and increased glycolysis after mercuric chloride induced-AKI^29^. Glycolysis derived methylglyoxal is associated with changes in kidney function among individuals with screen-detected Type 2 diabetes mellitus^30^.

Aberrant induction of glycolysis in autosomal dominant polycystic kidney disease was studied in which the defective glycolysis shares very similar features to aerobic glycolysis; treatment with glycolysis inhibitor 2-deoxyglucose (2-DG) suppressed the disease phenotype^31^. Here we hypothesized that kidney fibrosis in diabetes is associated with aberrant glycolysis and inhibition of such aberrant glycolysis is the potential therapeutic target to combat fibrosis associated with diabetic kidney disease.

## Results

### SIRT3 deficiency leads to fibrogenic phenotype in the kidney of diabetic mice

**Fig. 1:**
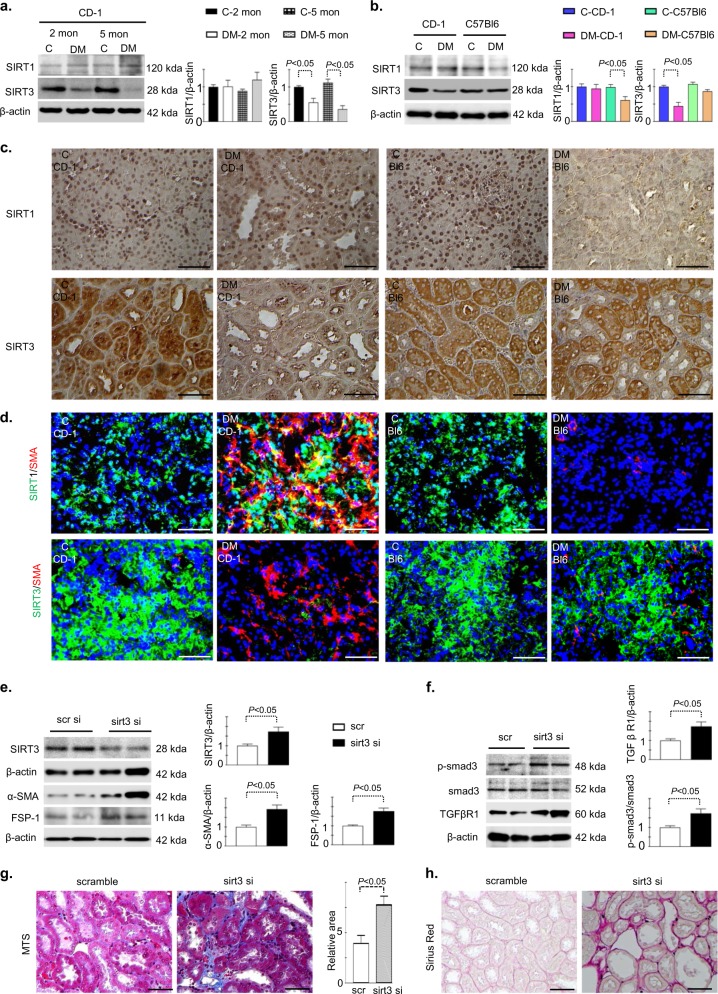
SIRT3 deficiency is associated with fibrogenic phenotype in the kidney of diabetic mice. **a** Western blot analysis of SIRT1 and SIRT3 protein in the time dependent STZ-induced diabetic kidney of CD-1 mice. Representative blots from three independent experiments are shown. Densitometric quantification by ImageJ program. **b** Western blot analysis of SIRT1 and SIRT3 protein in the control and streptozotocin (STZ)-induced diabetic kidney of CD-1 and C57Bl6 mice. The representative blots from six independent experiments are shown. **c** Immuno-histochemical analysis of SIRT1 and SIRT3 protein. The representative pictures are shown. **d** Immuno-fluorescence analysis was performed by fluorescence microscopy. FITC-labeled SIRT1 and SIRT3, Rhodamine-labeled α-SMA and DAPI blue. Merged images are shown. Scale bar: 50 μm in each panel. Representative pictures are shown. **e** Western blot analysis of SIRT3, α-SMA and FSP-1 in the diabetic kidney of scramble and sirt3 siRNA-injected mice. Scramble and sirt3 siRNA were injected into the tail vein twice in the week for 4 weeks at a dose of 5 mg/kg body weight. A representative image from five blots is shown. **f** Western blot analysis of smad3 phosphorylation and TGFβR1 in the diabetic kidney of scramble and sirt3 siRNA-injected mice. A representative image from five blots is shown. **g** Masson’s trichrome staining (MTS) in the diabetic kidney of scramble and sirt3 siRNA-injected mice and the quantification of the relative area fibrogenesis (RAF) by ImageJ. Scale bar: 50 µM. **h** Sirius red staining and quantification. Scale bar: 50 µM. *N* = 6 were analyzed in each group. Data are expressed as the mean ± SEM and are shown in the graph. Tukey test was performed to calculate statistical significance. C for control while DM depicts diabetic group

Streptozotocin (STZ)-induced diabetic CD-1 mice is the established mouse model for the study of diabetic kidney disease^32–34^. The kidney of STZ induced diabetic mice has been shown to display massive fibrosis in 6 month after a single injection of STZ^32–34^. Here we showed that the kidney of diabetic CD-1 mice displayed time dependent reduction in the SIRT3 protein whereas SIRT1 protein was not significantly altered (Fig. 1a). In mice, the kidney fibrosis phenotype is largely dependent upon the strain specificity^32–34^. We analyzed comparative renal histopathology in non-diabetic and diabetic CD-1 and C57Bl6 mice. At the time of sacrifice, the diabetic CD-1 and diabetic C57Bl6 mice had similar blood glucose levels (Supplementary Figure S1a), reduced body weight (Supplementary Figure S1b), higher kidney weight per body weight (Supplementary Figure S1c), higher plasma albumin creatine ratio (Supplementary Figure S1d) compared with the non-diabetic animals. However, kidney of diabetic CD-1 mice had relatively higher kidney weight, plasma albumin creatinine ratio and cystatin C level (Supplementary Figure S1c–e). The kidney of the diabetic CD-1 mice displayed massive fibrosis, with excessive deposition of the extracellular matrix (ECM) while the kidney of diabetic C57Bl6 mice showed minor fibrotic alterations. (Supplementary Figure S1f and g), as reported earlier^32^. Such fibrotic kidney of diabetic CD-1 mice exhibited suppression of the SIRT3 protein level which was not observed in the less-fibrotic kidney of diabetic C57Bl6 mice (Fig. 1b, c). SIRT1 protein level was found unaltered in the fibrotic kidney of diabetic CD-1 mice while suppressed in the less fibrotic kidney of diabetic C57Bl6 mice when compared to kidney of control mice (Fig. 1b, c). Intriguingly while analyzing SIRT1/α-SMA co-labeling, α-SMA positive cells displayed unremarkable alteration in the SIRT1 level in the kidney of diabetic CD-1 mice; whereas α-SMA positive cells showed the diminished level of the SIRT1 in the diabetic kidney of C57Bl6 mice (Fig. 1d). However, analysis of SIRT3/α-SMA co-labeling exhibited suppressed level in the kidney of diabetic CD-1 mice (Fig. 1d). Furthermore, to verify the role of SIRT3 in the fibrosis, we administered specifically designed SIRT3 siRNA into the tail vein of diabetic mice which had early onset of fibrosis (2 months post STZ induced diabetic mice). This procedure significantly suppressed the SIRT3 protein level in the kidney (Fig. 1e). Knockdown of SIRT3 in these mice did not cause any alterations either in the blood glucose level or in the body weight (Supplementary Figure S1h and i); however higher kidney weight per body weight, albumin creatinine ratio and Cystatin C level were observed (Supplementary Figure S1j–l). Importantly, knockdown of the SIRT3 resulted into the concomitant induction of fibrogenic markers, TGFβR1 and smad3 phosphorylation (Fig. 1e, f and Supplementary Figure S1m). Moreover, knockdown of SIRT3 accelerated the onset of fibrosis, exacerbated collagen and ECM deposition (Fig. 1g, h).

To probe the reason for robust SIRT3 deficiency in the diabetes-associated fibrosis in kidney, we analyzed peroxisome proliferator-activated receptor-gamma coactivator (PGC)-1α which is known to regulate SIRT3^35–37^. Indeed, we observed the suppressed PGC1α and carnitine O-palmitoyltransferase 1 (CPT1A) in the kidney of diabetic CD-1 mice (Supplementary Figure S1n).

Moreover, to test the role of SIRT1 in diabetes-associated kidney fibrosis, we injected sirt1 siRNA and scramble siRNA in the tail vein of 2 months post STZ induced diabetic mice. Sirt1 siRNA did not cause any remarkable difference in the body weight, blood glucose, kidney weight, albumin to creatinine ratio and in the cystatin C level (Supplementary Figure S2a). Sirt1 siRNA injection caused significant suppression in the protein level of SIRT1 but we did not observe any remarkable difference in the level of α-SMA, TGFβ and smad3 phosphorylation (Supplementary Figure S2b–f). We also analyzed the relative area fibrosis and collagen deposition but we could not get any significant difference (Supplementary Figure S2d, e).

To test for the cellular localization of SIRT1 and SIRT3, we performed cellular fractional analysis of kidney in control and in diabetic CD-1 mice. We did not observe the significant alterations in the protein level of SIRT1 in the nuclear fraction, whereas in the cytosolic SIRT1 level was induced in the diabetic CD-1 mice when compare to control. In the case of SIRT3, both mitochondrial and cytosolic SIRT3 was suppressed in the kidney of diabetic CD-1 mice.

SIRT1 protein level was significantly reduced in the nuclear fraction of diabetic kidneys of C57Bl6. However, we did not observe any remarkable alteration the protein level of SIRT3 in the cytosolic and mitochondrial fraction when compare to control C57Bl6 mice (Supplementary Figure S3a, b).

### Fibrotic phenotype exhibits induction of abnormal glucose metabolism in diabetic kidney

**Fig. 2:**
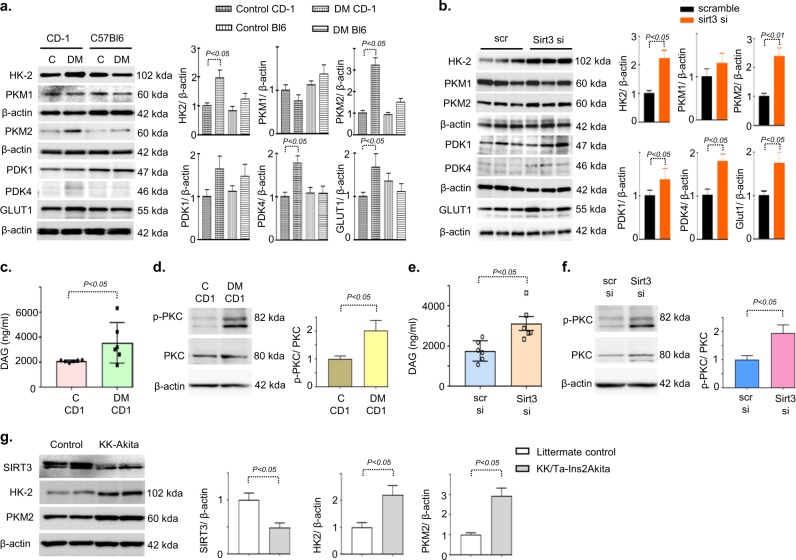
SIRT3 deficiency leads to induction of abnormal glycolysis. **a** Western blot analysis of HK2, PKM1, PKM2, PDK1, PDK4, and GLUT1, in the control and diabetic kidneys of CD-1 and C57Bl6 mice. Representatives from five blots are shown. Densitometry quantifications were normalized by β-actin. **b** Western blot analysis of HK2, PKM1, PKM2, PDK1, PDK4, and GLUT1, in the scramble, injected and sirt3 siRNA tail vein injected kidneys of diabetic CD-1 mice. Representative from four blots is shown. Densitometric quantifications were normalized by β-actin. **c** DAG level was analyzed by Elisa assay. *N* = 6 were analyzed in the kidneys of control and diabetic CD-1 mice. **d** Western blot analysis of PKC phophorylation and PKC were analyzed in the kidneys of control and diabetic CD-1 mice. The representative from four blots is shown. **e** DAG level was measured by Elisa assay. *N* = 6 were analyzed in the kidneys of scramble siRNA and sirt3 siRNA injected mice. **f** Western blot analysis of PKC phophorylation and PKC were analyzed in the kidneys of scramble siRNA and sirt3 siRNA injected mice. The representative from four blots is shown. **g** Western blot analysis of SIRT3, HK-2 and PKM2 in the kidneys of littermate control and KK/Ta-Ins2Akita mice. The representative from four blots is shown. Densitometric quantifications were normalized by β-actin. Data in the graph are expressed as mean ± SEM. Tukey test was performed to calculate statistical significance

Further, we aimed to explore the role of SIRT3 suppression and its association with glucose metabolism in kidney fibrosis. We analyzed the protein expression of the key enzymes of glycolysis in the fibrotic kidney of diabetic CD-1 and less fibrotic kidney of diabetic C57Bl6 mice. Western blot and immunohistochemical analysis revealed induction of glucose transporter 1 (GLUT1), hexokinase 2 (HK2), pyruvate kinase M2 type (PKM2) and pyruvate dehydrogenase kinase 4 (PDK4) protein expression in the damaged tubular area (Fig. 2a and Supplementary Figure S4a). Moreover, SIRT3 knockdown in the kidney of diabetic CD-1 mice caused induction of HK2, PKM2, and PDK4 protein expression (Fig. 2b). We did not observe any remarkable alteration in the protein level of GLUT1, HK2, PKM2, and PDK4 after sirt1 knockdown in the kidney of diabetic CD-1 mice (Supplementary Figure S4b).

Furthermore, we analyzed the DAG accumulation and activation of PKC in the kidney of control and diabetic CD-1 mice. We observed remarkable increase in the level of DAG accumulation and induction of PKC phosphorylation (Fig. 2c, d). SIRT3 knockdown displayed DAG accumulation and induction of PKC phosphorylation (Fig. 2e, f).

Further, we confirmed our finding in the genetic model of diabetic nephropathy, i.e., KK/Ta-Ins2(Akita) diabetic mice, which exhibit progressive diabetic kidney disease^38^. We observed the suppression of SIRT3 protein level and induction of HK2 and PKM2 when compare to littermate control, suggesting that suppression of SIRT3 is associated with the induction of key glycolysis enzymes which could be key fibrotic phenotype in the mice (Fig. 2g).

### Abnormal glycolysis accelerates mesenchymal transformations, and myofibroblasts O-linked glycosylation

**Fig. 3:**
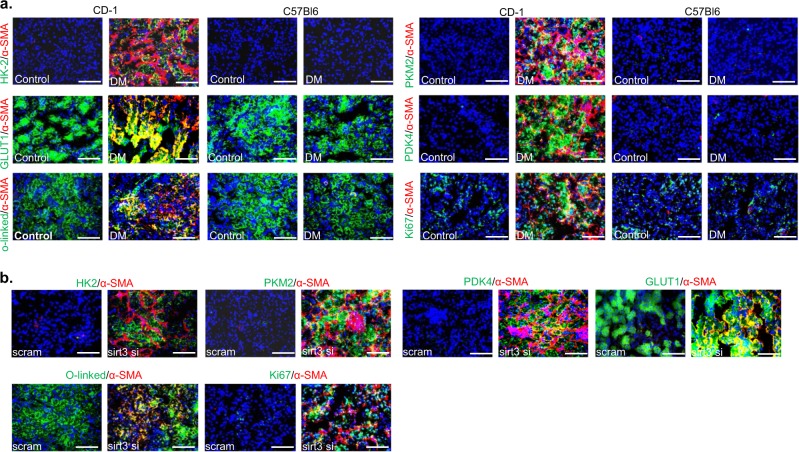
Abnormal glycolysis is linked with myofibroblasts formation, proliferation, and O-linked glycosylation. **a** Co-immunolabelling of HK2, PKM2, GLUT1, PDK4, proliferation marker ki67 and O-linked glycosylation with α-SMA, were analyzed by using fluorescence microscope. *N* = 3 were analyzed in the case of control while *n* = 5 were analyzed in the diabetic group of both strains. Representative figure in each panel is shown. **b** Co-immunolabelling of glycolytic enzymes, proliferation marker ki67 and O-linked glycosylation with α-SMA were analyzed using fluorescence microscope in the diabetic kidney of scramble and sirt3 siRNA injected mice. Scramble *N* = 3, Sirt3 siRNA *n* = 4 were analyzed. Representative figure in each panel is shown

Increased rate of mesenchymal transformation in the diabetic kidney has been identified as one of the mechanisms causing fibrosis and such mesenchymal cells reprogram their metabolism and depend on glycolytic metabolites for nucleic acid, amino acids, glycoproteins, glycerol, and lipid synthesis^39^. We attempted to find out the correlation between SIRT3 deficiency-linked-abnormal glycolysis and mesenchymal activation in the fibrotic kidney. The Higher level of gene expression of mesenchymal transcriptional inducers such as TGFβ1 and snail1 were observed in fibrotic kidney (Supplementary Figure S5a). These mesenchymal inducers are known as the repressor of E-cadherin and elevate the level of mesenchymal markers such as α-SMA and vimentin, drive the renal fibrosis^39^. To test the contribution of abnormal glycolysis in the induction of α-SMA, we performed co-immunolabeling of glycolytic enzymes with α-SMA. Higher level of HK2/α-SMA, PKM2/α-SMA, PDK4/α-SMA, GLUT1/α-SMA, proliferation marker Ki67/α-SMA and O-linked/α-SMA whereas α-SMA positive cells displayed no alterations in the level of hexokinase 1 (HK1), pyruvate kinase M1 type 1 (PKM1) and pyruvate dehydrogenase kinase 1 (PDK1) co-immunolabeling in the fibrotic kidney of diabetic CD-1 mice (Fig. 3a and Supplementary Figure S5b) suggesting that induction of HK2, PKM2, and PDK4 was associated with mesenchymal transformations, myofibroblasts proliferation and O-linked glycosylation. SIRT3 knockdown in kidney of diabetic mice displayed higher expression level of mesenchymal inducers (Supplementary Figure S5c) and higher co-labeling of HK2/α-SMA, PKM2/α-SMA, PDK4/α-SMA, GLUT1/α-SMA, Ki67/α-SMA and O-linked/α-SMA co-immunolabeling when compared to the diabetic kidney of scramble (Fig. 3b), suggesting that SIRT3 deficiency leads to the induction of abnormal glycolysis and associated-mesenchymal transformations.

### Glycolysis inhibitions cancel SIRT3 deficiency linked abnormal glucose metabolism and ameliorate renal fibrosis

**Fig. 4:**
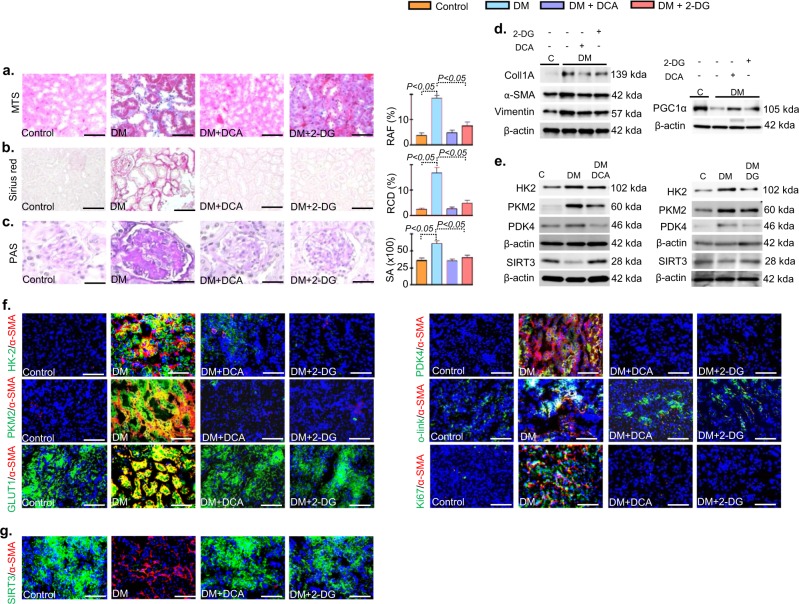
Glycolysis inhibitions suppress fibrogenic phenotypes and aberrant glycolysis associated injury. **a** MTS, **b**, Sirius red, **c** PAS in the control, diabetic, dichloroacetate (DCA) and 2-deoxyglucose (2-DG) treated diabetic kidney. Quantification of the relative area fibrogenesis (RAF), relative collagen deposition (RCD) and surface area (SA) was performed by ImageJ. Scale bar: 50 µM Control *n* = 5, Diabetic *n* = 6, DCA *n* = 7 and 2-DG *n* = 3, were analyzed. The representative picture in each panel is shown. **d** Western blot analysis of Colla1A, α-SMA, Vimentin, and PGC-1α was analyzed. Representative blots from five independent sets have been shown. *N* = 5 were analyzed in each group. **e** Western blot analysis of HK2, PKM2, PDK4, and SIRT3 was analyzed in control diabetes and DCA and 2-DG treated diabetic kidney. Representative blots from five independent sets are shown. *N* = 5 were analyzed in each group. **f, g** Co-immunolabelling of HK2, PKM2, PDK4, and GLUT1, ki67, O-linked glucosylation and SIRT3 with α-SMA were analyzed using fluorescence microscope. Control *n* = 3, DM *n* = 4, DCA *n* = 5 and 2-DG *n* = 3 were analyzed. Representative figure in each panel is shown. Data in the graph are expressed as the mean ± SEM. Tukey test was performed to calculate statistical significance

To study the glycolysis inhibition in renal fibrosis we utilized two kind of inhibitors: (1) 2-deoxyglucose (2-DG), which is a physiological inhibitor of glycolysis blocks the phosphorylation step of glucose and (2) dichloroacetate (DCA), which block the anaerobic glycolysis/Warburg metabolism, by inhibiting PDKs (Supplementary Figure S6). At the time of sacrifice, DCA and 2-DG treatment did not alter the blood glucose (Supplementary Figure S7a and b); DCA caused increase in the systolic and diastolic blood pressure while 2-DG only caused increase in diastolic blood pressure (Supplementary Figure S7c and d); no remarkable change in body weight whereas, reduction in kidney weight per body weight, in albumin creatinine ratio and in cystatin C level was observed (Supplementary Figure S7e–l). Both DCA and 2-DG suppressed the ECM deposition, collagen accumulation and glomerulosclerosis (Fig. 4a–c). DCA and 2-DG treatment reduced the protein levels of mesenchymal markers (α-SMA, vimentin, and coll1A), HK2, PKM2, PDK4 with concomitant induction in the protein level of SIRT3 and PGC1-α (Fig. 4d, e and Supplementary Figure S7m and n). Glycolysis inhibitions suppressed the higher level of HK2/α-SMA, PKM2/α-SMA, PDK4/α-SMA, GLUT1/α-SMA, Ki67/α-SMA and O-linked/α-SMA co-immunolabeling (Fig. 4f) whereas restored the SIRT3 protein in the diabetic mice (Fig. 4g).

Moreover, we also analyzed the efficacy of DCA and 2-DG in the diabetic C57Bl6 mice. The treatment of DCA and 2-DG did not cause any remarkable difference in the body weight, blood glucose, kidney weight and in relative area fibrosis when compare to diabetic group (Supplementary Figure S8a). DCA treatment caused restoration of SIRT1 protein level while we did not observe any remarkable difference in the SIRT3 protein level when compare to diabetic group (Supplementary Figure S8b).

DCA and 2-DG treatment caused suppression in the protein level of SIRT1 in the diabetic CD-1 mice (Supplementary Figure S8c).

### SIRT3 deficiency leads to abnormal glycolysis in vitro and glycolysis inhibition by dichloroacetate require SIRT3 for its action

**Fig. 5:**
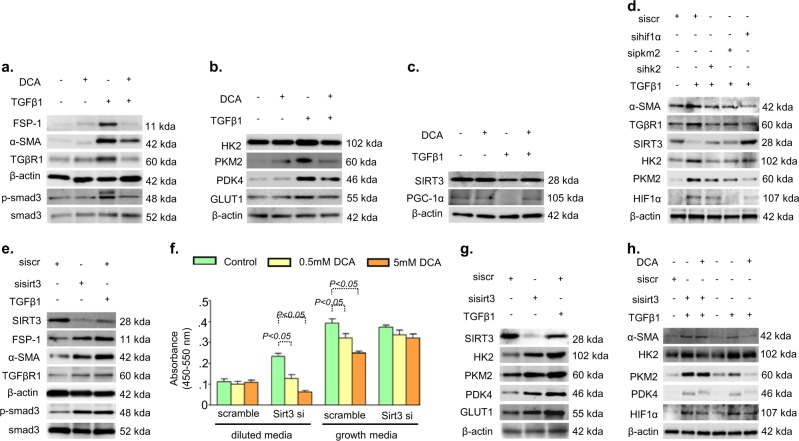
Glycolysis inhibition suppresses the fibrogenic characteristics and associated abnormal glycolysis in vitro. **a**–**c** Western blot analysis of FSP-1, α-SMA, TGFβR1, smad3 phosphorylation, total smad3, HK2, PKM2, PDK4, GLUT1, SIRT3, and PGC-1α was performed in the DCA treated cells which were incubated with (10 ng/ml) or without TGFβ1 treatment in HK-2 cells. **d** Western blots analysis of α-SMA, TGFβR1, HK2, PKM2, SIRT3 and HIF1α in the hk2 siRNA, pkm2 siRNA and hif1α siRNA transfected HK-2 cells. Representative blots from five independent experiments are shown. **e** Western blots analysis of SIRT3, FSP-1, α-SMA, TGFβR1, smad3 phosphorylation and total smad3 in the scramble siRNA and sirt3 siRNA transfected HK-2 cells. Representative blots from four independent experiments are shown. **f** BrdU cell proliferation assays were performed in the DCA treated (at 0.5 and 5 mM concentration) scramble siRNA and sirt3 siRNA transfected HK-2 cells, in the growth media and diluted media. Samples in triplicate were analyzed. **g** Western blot analysis of SIRT3, HK2, PKM2 PDK4 and GLUT1 in the scramble and sirt3 siRNA transfected HK-2 cells. The representative from four blots is shown here. **h** Western blot analysis of α-SMA, HK2, PKM2, PDK4, and HIF1α in the DCA treated scramble and sirt3 siRNA transfected HK-2 cells. Three independent sets were analyzed. Data in the graph are expressed as mean ± SEM. Tukey test was performed to calculate statistical significance

To confirm the results in vivo, we utilized the human proximal tubular epithelial cells (HK-2 cells). To define glycolysis pathways specific to TGFβ1, we examined protein expression analysis of glucose metabolism enzymes in the HK-2 cells. TGFβ1 treatment caused induction of FSP-1, α-SMA, TGFβR1, smad3 phosphorylation, HK2, PKM2, PDK4 and GLUT1 whereas suppressed the protein levels of SIRT3, PGC1α, and CPT1A (Fig. 5a–c and Supplementary Figure S9a–d). DCA treatment abolished such effects (Fig. 5a–c and Supplementary Figure S9a–d).

SIRT3 destabilizes HIF1α induced metabolic reprogramming in cancer cells^40–43^. To examine the role of HIF1α in fibrogenesis, we knocked down hk2, pkm2, and hif1α by using specific siRNAs in presence of TGFβ1. Silencing of hk2, pkm2, and hif1α eliminated the TGFβ1-induced gain of α-SMA with concomitant induction of SIRT3 protein level, suggesting that TGFβ1-derived fibrogenesis was linked to SIRT3 deficiency associated induction HIF1α inducible enzymes (Fig. 5d and Supplementary Figure S9e). Silencing of SIRT3 by siRNA demonstrated induction of mesenchymal markers, TGFβR1 protein and smad3 phosphorylation (Fig. 5e and Supplementary Figure S9f). To study the impact of glycolysis on SIRT3 deficient cells, we treated the SIRT3 siRNA-transfected cells with DCA in the growth media and diluted media. Inhibition of glycolysis caused suppressed cell proliferation in diluted media, suggesting that glycolysis was necessary for the growth of SIRT3 deficient cells (Fig. 5f). Such dependence on glycolysis was associated with the induction of glycolysis at the protein level (Fig. 5g and Supplementary Figure S9f). Glycolysis inhibition suppressed the HK2, PDK4, HIF1α and GLUT1 protein levels but unable to suppress PKM2 and αSMA in the SIRT3 siRNA-transfected cells, confirming that DCA requires SIRT3 protein for its action on the suppression of PKM2 and PKM2-associated induction of mesenchymal features in HK-2 cells (Fig. 5h and Supplementary Figure S9g).

**Fig. 6:**
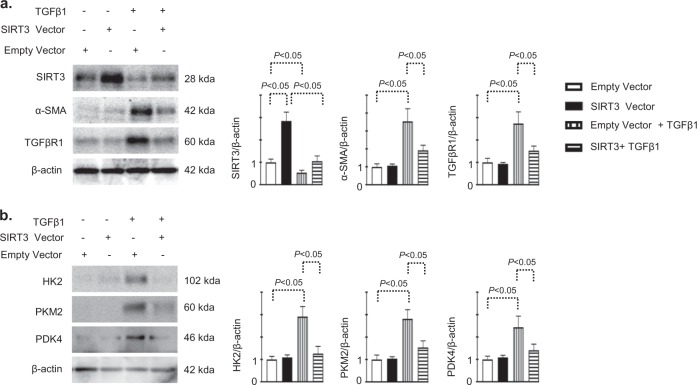
Over-expression of SIRT3 protein leads to suppression of TGFβ2-associated fibrogenic features in HK2 cells. **a**, **b** Western blot analysis of SIRT3, α-SMA, TGFβR1, HK2, PKM2 and PDK4 in the empty vector and SIRT3 vector transfected HK-2 cells-treated with or without TGFβ1. The representative from four blots is shown. Densitometric quantifications were normalized by β-actin. Data in the graph are expressed as mean ± SEM. Tukey test was performed to calculate statistical significance

To study the SIRT3 over-expression and its association with anti-fibrogenic features, we transfected the control vector and sirt3 over expression vector in the presence and absence of TGFβ1 in the HK-2 cells. SIRT3 over-expression vector transfection caused significant induction of SIRT3 protein level in the absence and presence of TGFβ1 (Fig. 6a). Over-expression of SIRT3 resulted into suppression of TGFβ1 stimulated-mesenchymal activation and protein level of HK2, PKM2 and PDK4 (Fig. 6b). Moreover, we found higher level of VEGF proetin in the kidney of diabetic CD-1 mice when compare to control and SIRT3 knockdown desplayed induction of VEGF protein level in the diabetic kidney (Supplementary Figure S10a and b).

### SIRT3 deficiency leads to accumulation of HIF1α and induction of PKM2 dimer formation

**Fig. 7:**
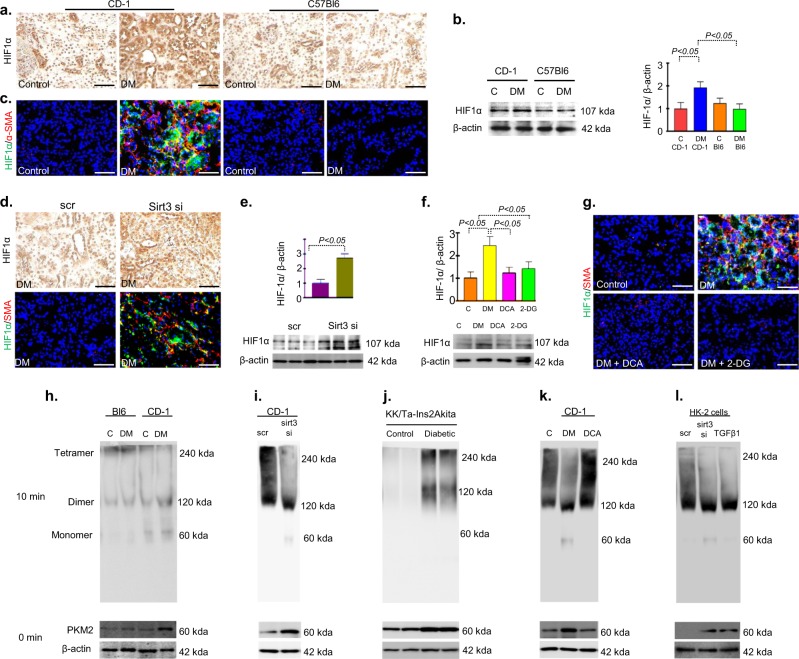
Cumulative effects of HIF1α accumulation and PKM2 dimer formation lead to aberrant glycolysis during fibrotic events. **a** Immunostaining of HIF1α. **b** Western blot analysis of HIF1α in the control and diabetic kidney of CD-1 and C57Bl6 mice strains. Representative blot from four blots is shown. **c** Co-immunolabelling of HIF1α with αSMA was analyzed by fluorescence microscope. Green FITC-HIF1α, Red Rhodamine-αSMA. Representative pictures are shown. Scale bar 50 µM. **d** Immunostaining of HIF1α in the scramble and sirt3 siRNA injected diabetic kidney. Representative pictures are shown. **e** Western blot analysis of HIF1α in scramble and sirt3 siRNA injected diabetic kidney. Representative blot from three blots is shown. **f** Western blot analysis of HIF1α in kidney of control, diabetic, DCA and 2-DG treated diabetic CD-1 mice. Representative blot from four blots is shown. **g** Co-immunolabelling of HIF1α with αSMA in the kidney of control, diabetic, DCA and 2-DG treatment in diabetic CD-1 mice. Scale bar 50 µM. Green FITC-HIF1α, Red Rhodamine-αSMA. Representative pictures are shown. **h** Glutaryldehyde chemical cross-linking experiment was performed in the kidney of control and diabetes of CD-1 and C57Bl6 mice strains. The representative from four blots is shown. *N* = 4 were evaluated in each group. **i** In the scramble and sirt3 siRNA transfected diabetic CD-1 mice. *N* = 5 were evaluated. **j** In the kidneys of littermate control and diabetic KK/Ta-Ins2Akita mice. **k** In control, diabetes, and DCA treated diabetic CD-1 mice. *N* = 5 were analyzed in each group. **l** In the scramble, sirt3 siRNA transfected and TGFβ alone treated HK-2 cells. *N* = 4 were analyzed in each group. Data in the each graph are shown as mean ± SEM. Tukey test was performed for statistical significance

Our results exhibited accumulation of HIF1α protein in the fibrotic kidney of diabetic CD-1 mice (Fig. 7a, b). Immunofluorescence data revealed the higher level of co-immunolabeling of HIF1α/α-SMA in the fibrotic kidney of diabetic CD-1 mice (Fig. 7c). Also in the kidney of SIRT3 knockdown diabetic mice revealed higher level of HIF1α accumulation and induced level of co-labeling of HIF1α/α-SMA (Fig. 7d, e). Glycolysis inhibitions led to reduce the HIF1α protein level and HIF1α/α-SMA co-immunolabeling (Fig. 7f, g).

**Fig. 8:**
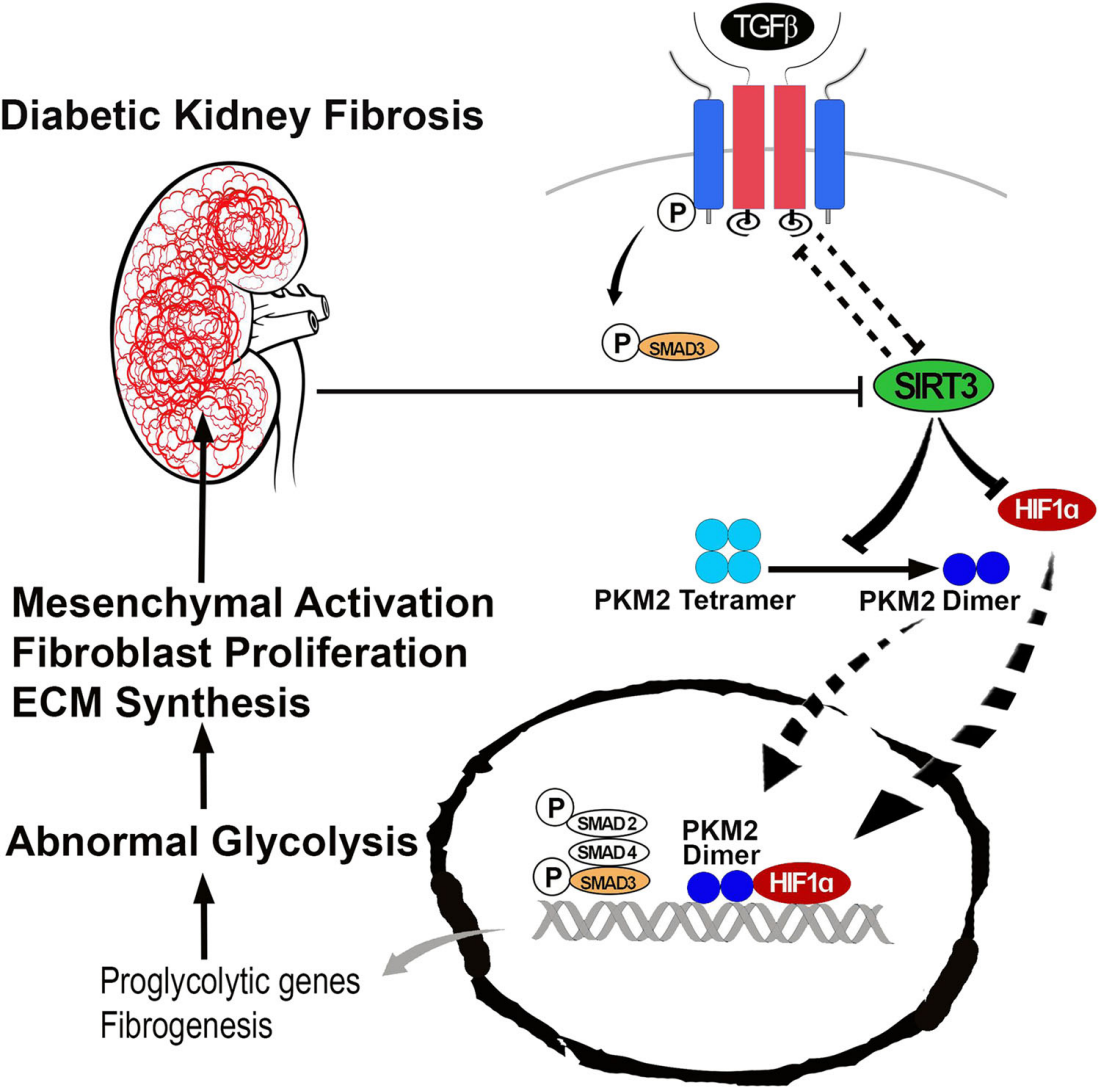
The working hypothesis showing SIRT3 suppression associated activation of the mesenchymal program and renal fibrosis through induction of abnormal glycolysis by modulating the HIF1α accumulation and increase PKM2 dimer formation

PKM2 resides in the two forms, tetramer and dimer. Tetramer form has catalytic activity transfer phosphoryl group from PEP to ADP and produces ATP and pyruvate whereas PKM2 dimer translocates into the nucleus, regulate HIF1α and IL-1β production, and induce the activation of pro-glycolytic enzymes during the inflammation and tumorigenesis^44–46^. Our data displayed the induction of PKM2 dimer formation and suppression of tetramer in the fibrotic kidney of diabetic mice (Fig. 7h). Additionally, we also observed induction of PKM2 dimer formation and suppression of tetramer in the fibrotic kidney of SIRT3 siRNA injected diabetic mice (Fig. 7i). Moreover, the kidneys of KK/Ta-Ins2Akita mice, the mouse model of progressive diabetic kidney disease^38^, displayed higher level of PKM2 dimer as compared with their littermate non-diabetic control (KK/Ta-wild-type) mice (Fig. 7j). Glycolysis inhibition suppressed the dimer form and induced the tetramer form (Fig. 7k). SIRT3 siRNA transfected HK-2 cells displayed suppression of tetramer and induced the dimer formation (Fig. 7l). Fig. 8 depicts the schematic diagram showing that the SIRT3 deficiency-associated abnormal glycolysis in fibrotic phenotypes was linked with PKM2 dimer formation and suppression of tetramer, such tetramer-to-dimer switching is the crucial in the pathobiology of diabetes associated kidney fibrosis.

## Discussion

Our results demonstrate that in diabetic kidney the SIRT3 deficiency led to induction of abnormal glycolysis through induction of TGFβ-smad3 signaling pathway. SIRT3 knockdown in mice which had early onset of fibrosis and in the cultured cells showed gain of mesenchymal features with induction of TGFβ-smad3 signaling, suggesting that SIRT3 suppression is an important step in the induction of TGFβ-smad3 signaling^13,16^. During our analysis, we also observed a SIRT3 deficiency-associated exacerbation of glycolysis, suggesting that the SIRT3 deficiency and subsequent induction of TGFβ signaling was associated with abnormal glycolysis. In addition, such induction of abnormal glycolysis was associated with an increased rate of mesenchymal transformations. Similarly, the clinical data suggests that in the diabetic subjects with chronic kidney disease, the oxygen consumption remains elevated with higher lactate level in the kidney and increase rate of glycolysis^24^.

Kidney epithelial cells require high base line of energy because of its functions. It utilizes the free fatty acids as the energy source. The damaged kidney epithelial cells exhibit defective fatty acid oxidation^28^. The glucose metabolism also participates in the fibrotic events to fulfill the metabolic requirement of myofibroblasts undergoing transformations^45–48^. The upregulated level of glycolysis-induced production of diacylglycerols is known to be key molecule for activation of protein kinase C-induced pathways, including the production of VEGF, TGFβ1, PAI-1, and NFҟB signaling, contributes in the development of diabetic kidney disease^49–52^. It further strengthened our views that induced level of glycolysis could be one of the adaptive mechanisms of fibroblasts survival and proliferation which contributes to development of fibrotic events in diabetes. Our results indicate that the inhibition of glycolysis suppressed the fibrogenic characteristics, abnormal glycolysis, mesenchymal transformations and restored SIRT3 protein level, confirming that abnormal glycolysis was associated with SIRT3 deficiency and contributes in the fibrotic events.

SIRT3 deficiency was associated with the suppression in PGC1α which is known to regulate renal oxidative metabolism in the epithelium^37^. Smad3 directly binds to the regulatory region of PGC1α and suppresses mitochondrial biosynthesis and metabolism^53^. Suppression of PGC1α in the fibrogenic phenotype explains the phenomenon of SIRT3 deficiency in the diabetic kidney of fibrotic strain^35,36^. Inhibition of abnormal glycolysis induced the PGC1α level, therefore, restored the SIRT3 level, suggesting that PGC1α-mediated SIRT3 restoration contributes to maintaining the homeostasis between glycolysis and mitochondrial metabolism.

Further glycolytic inhibition by DCA resulted in the suppression of TGFβ1-stimulated induction of PKM2 while glycolytic inhibition did not suppress PKM2 in SIRT3 deficient cells, suggesting that SIRT3 is the essential protein for the action of DCA. It is evident from the results that the higher level of glycolytic proteins was due to induce level of PKM2 dimer formation and suppress level of PKM2 tetramer. PKM2 dimer is the inactive form, enhances the transcription of pro-glycolytic enzymes, which leads to the accumulation of glycolytic intermediates above pyruvate kinase and these metabolites are then channeled into the nucleic acid, phospholipids, glycoprotein, amino acids, O-linked glycosylation synthesis^43,44,54–56^. The cumulative effects of PKM2 dimer formation and HIF1α accumulation mediate the induction of defective glycolysis. Our data is in accord with recent studies showing that activation of PKM2 tetramer may protect against the progression of diabetic glomerular pathology and mitochondrial dysfunction^57^.

In conclusion, our finding switches to the SIRT3 deficiency which leads to induction of abnormal glycolysis by activation of PKM2 dimer formation and HIF1α accumulation. Such abnormal glycolysis accelerates metabolic reprogramming in the injured epithelial cells of the fibrotic kidney. Glycolysis inhibition disrupts such metabolic reprogramming with significant suppression of fibrosis. This study provides a new therapeutic approach in diabetic kidney disease.

## Materials and methods

### Reagent and antibodies

Rabbit polyclonal, anti-PKM1, PKM2, HK-1 and PDK-1 antibodies were purchased from Cell Signaling Technology (Danvers, MA). A mouse monoclonal anti-α-SMA antibody was purchased from Life Span Biosciences, Inc Seattle WA. Mouse monoclonal anti-HIF-1α, rabbit polyclonal anti-GLUT1 and PDK4, mouse monoclonal O-linked N-acetyl glucosamine, rabbit PKC (phosphoT497) and rabbit PKC (ab19031) were purchased from Abcam (Cambridge, UK). A rabbit polyclonal anti-phospho smad3 (s423 and s425) antibody was purchased from Rockland Immunochemicals (Gilbertsville, PA). Mouse VEGF was purchased from RD System. A rabbit polyclonal anti-αSMA antibody was purchased from GeneTex (Irvine, CA). Rabbit polyclonal anti-TGF-βR1 antibody, mouse monoclonal anti-β-actin antibody, and rabbit polyclonal anti-GAPDH antibodies were obtained from Sigma (St Louis, MO). A mouse specific rabbit polyclonal anti-SIRT1 and goat polyclonal anti-SIRT3 antibody, anti-Ki67 was purchased from Santa Cruz Biotechnology (Dallas, TX). Anti-fibroblast specific proteins (FSP1, sometimes displayed as S100A4) was purchased from Biolegend CA. Fluorescence-, Alexa Fluor 647-, and rhodamine conjugated secondary antibodies were obtained from Jackson ImmunoResearch (West Grove, PA). A horseradish peroxidase-conjugated secondary antibody and TGFβ1 was purchased from PeproTech (Rocky Hill, NJ).

### Animal experimentation

The experiments in the methods sections are carried out in accordance with Kanazawa Medical University animal protocols (protocol number 2014–89; 2013–114 and 2014–101). KK/Ta-Ins2Akita mouse experiments were performed in accordance with the Animal Welfare Guidelines of Akita University and approved by the committee on Animal Experimentation of Akita University. Authors confirm that all the experiments are performed in accordance to Japanese guidelines and regulations for scientific and ethical experimentation. The induction of diabetes in the CD-1 and C57Bl6 KsJ mice was performed according to the previously established experimental protocol^32–34,58,59^. In brief, 8-week-old CD-1 and C57Bl6KsJ mice were induced diabetes with the single intraperitoneal injection of streptozotocin (STZ) at 200 mg/kg in 10 mmol/l citrate buffer (pH 4.5). Cystatin C levels in plasma were analyzed using the Mouse/Rat cystatin C kit (R&D System). Urine albumin levels were estimated using a Mouse Albumin ELISA Kit (Exocell, Philadelphia, PA). For the interventional study, we utilized a fibrotic diabetic kidney disease model (STZ-treated CD-1 mice). Twenty weeks after the induction of diabetes, the diabetic mice were divided into the following three groups: a control group, dichloroacetate (DCA) treatment group (1 g/L body weight/day in drinking water) and 2-deoxyglucose (2-DG) treatment group for 500 µg/kg i.p. twice in a week for 4 weeks.

Kidneys were harvested from the 15-week-old KK/Ta-Ins2Akita mice and their littermate non-diabetic control (KK/Ta-wild-type) mice to confirm our findings. The kidneys were perfused via left ventricle with phosphate-buffered saline, and then stored at −80 °C until western blot analysis.

### In-vivo silencing studies by using SIRT3 siRNA

For the sirt3 in vivo knockdown study, we utilized diabetic CD-1 mice which have experienced 8 weeks of induction of diabetes, were divided into two groups: scramble group and Sirt3 siRNA group. A chemically modified HPLC purified sirt3 siRNA duplex (Sense strand 5′GUCUGAAGCAGUACAGAAAtt and antisense strand 5′UUUCUGUACUGCUUCAGACaa) and scramble siRNA duplex was purchased from Invitogen inVivo ready siRNAs. For SIRT1 knockdown study we purchased the duplex from Invitogen inVivo ready siRNA. All of the oligos were dissolved in buffer (Atelo gene, Koken Co. Ltd. Japan) and injected into the tail vein (100 μl) twice weekly for 3 weeks at the dose of 5 mg/kg body weight. For SIRT1 knockdown study we purchased the duplex from Invitogen inVivo ready siRNA.

### Morphological evaluation

We utilized a point-counting method to evaluate the relative area of the mesangial matrix. We analyzed PAS-stained glomeruli from each mouse using a digital microscope screen grid containing 540 (27 × 20) points. Masson’s trichrome-stained and Sirius red stained tissue images were evaluated by ImageJ software, and the fibrotic areas were estimated. For each mouse, images of six different fields of view were evaluated at 40x magnification.

### In vitro experiment

Human HK-2 cells was cultured in DMEM and Keratinocyte-SFM (1×) medium, (Life Technologies Green Island NY) medium respectively. When the cells on the adhesion reagent reached 70% confluence, 10 ng/ml recombinant human TGFβ1 for 48 h was placed in the serum diluted medium with or without DCA (100 nM) and 2-DG (100 nM) pre-incubation for 2 h.

### Western blot analysis

Protein lysates were denatured in the SDS sample buffer at 100 °C for 5 min and were separated on SDS-polyacrylamide gels and blotted onto PVDF membranes (Pall Corporation, Pensacola, FL, USA) using the semi-dry method. The immunoreactive bands were developed using an enhanced chemiluminescence (ECL) detection system (Pierce Biotechnology, Rockford, IL, USA) and detected using an ImageQuant LAS 400 digital biomolecular imaging system (GE Healthcare Life Sciences, Uppsala, Sweden).

### Chemical cross-linking experiment

Kidney tissues were lysed with sodium phosphate buffer (pH 7.3) containing 0.5% Triton X-100 and 1 × protease inhibitors (Thermo) for 30 min at 4 °C. Crude lysates were clarified by centrifugation at top speed (16, 000 × *g*) for 30 min at 4 °C. For carrying out crosslinking reactions, the supernatants (4 mg/mL) were treated with 2.3% or glutaraldehyde to the lysate at the final concentration of 5%. The lysate was then incubated at 37 °C for 10 min. The reactions were terminated by adding 1 M Tris buffer to a final concentration of 50 mM Tris·Cl (pH 8.0). The samples were mixed with 2 sample loading buffer and heated at 100 °C for 5 min. Samples were then separated by 5−20% SDS/PAGE and analyzed by Western blotting.

### Transfection

The HK-2 cells were transfected with 100 nM of specifically designed siRNA for hk-2, pkm2, glut-1 and hif1α using Lipofectamine 2000 transfection reagent (Invitrogen, Carlsbad, CA, USA), according to the manufacturer’s instructions. In the second set of experiment, we transfected specific sirt3 siRNA (Invitrogen, Carlsbad, CA, USA) at the concentration of 100 nM in the cells. Cells were treated with or without TGFβ1 (10 ng/ml) for 72 h, cells were harvested for western blot analysis. In the third set of experiment, we transfected specific sirt3 vector^60^ and empty vector at the concentration of 100 nM in the cells. Cells were treated with or without TGFβ1 (10 ng/ml) for 72 h, cells were harvested for western blot analysis.

### Statistical analysis

The data were expressed as the means ± S.E.M. One way Anova (Tukey test) was used for detect statistical significance (defined as *P* < 0.05). GraphPad prism 5.0 was used for statistical analysis.

## Electronic supplementary material

Supplemental Materials
