# Supplementary material for: SIRT3 deficiency leads to induction of abnormal glycolysis in diabetic kidney with fibrosis
Source: Cell Death Dis. 2018 Sep 24;9(10):997. doi: 10.1038/s41419-018-1057-0 (PMC6155322; doi:10.1038/s41419-018-1057-0)
Supplement: Supplementary file 1 — Supplemental Materials [file 41419_2018_1057_MOESM1_ESM.doc]

**SUPPLEMENTAL MATERIALS**

**SIRT3 deficiency leads to induction of abnormal glycolysis in Diabetic Kidney with fibrosis**

Swayam Prakash Srivastava^##^,^1,3^ Jinpeng Li^##^,^1^ Munehiro Kitada,^1,2^ Hiroki Fujita,^4^ Yuichiro Yamada,^4^ Julie E. Goodwin^3^, Keizo Kanasaki,^1,2^ and Daisuke Koya,^1,2^

1. Department of Diabetology & Endocrinology, Kanazawa Medical University, Uchinada, Ishikawa, Japan 920-0293
2. Division of Anticipatory Molecular Food Science and Technology, Kanazawa Medical University, Uchinada, Ishikawa, Japan 920-0293
3. Department of Pediatrics (Nephrology) Yale University School of Medicine, New Haven, CT, United States 06520
4. Department of Endocrinology, Diabetes and Geriatric Medicine, Akita University Graduate School of Medicine, Akita, Japan 010-8543

**Running Title: SIRT3 deficiency linked abnormal glycolysis**

##: equal contribution

***: Co-corresponding authors**

Address correspondence to:

**Keizo Kanasaki**

E-mail: [kkanasak@kanazawa-med.ac.jp](mailto:kkanasak@kanazawa-med.ac.jp)

or

**Daisuke Koya**

E-mail: [koya0516@kanazawa-med.ac.jp](mailto:kkanasak@kanazawa-med.ac.jp)

Department of Diabetology & Endocrinology

Kanazawa Medical University

Uchinada, Ishikawa 920-0293, Japan

TEL: 81-76-286-2211(Ex3305)

FAX: 81-76-286-6927

**Materials and Methods**

**RNA isolation and qPCR**

Frozen kidney tissues were first placed on the RNAlater®-I (Life technologies) for 16 h at -20 °C before the subsequent homogenization process. Total RNA was isolated using the RNeasy mini kit (Qiagen) following the manufacturer’s instructions and was quantified with a Nanodrop spectrophotometer (ND-1000, Nano drop Technologies, DE, USA). Complementary DNA (cDNA) was generated by RT kit (Takara Bio Inc.) using the concentration of 500ng/µl mRNA. mRNA gene expression was quantified by using SYBR green PCR kit (Takara Bio Inc.) Following are primers for quantification TGFβ1 and snail1.

| Gene name | Forward primer | Reverse primer |
| --- | --- | --- |
| TGFβ1 | 5’ AAAACCAAAGACATCTCACAC | 5’ GAATCGAAAGCCCTGTATTCC |
| Snail1 | 5’ CCGGAAGCCCAACTATAGCGA | 5’ TTCAGAGCGCCCAGGCTGAGGTACT |
| 18s | 5’CGAAAGCATTTGCCAAGAAT | 5’AGTCGGCATCGTTTATGGTC |

**Supplementary Figure 1 Physiological characteristics of control and diabetic mice of CD-1 and C57Bl6 mice**

**a.** Blood glucose level was evaluated in the control, and diabetic group of CD-1 and C57Bl6 mice strain before the sacrifice of mice. **b.** Body weight. **c.** Kidney weight (mg/g body weight). **d.** Albumin to creatinine ratio. **e.** Cystatin C (pg/ml). N=6 were analyzed in each group. **f-g.** Sirius red and Masson trichome stating (MTS) were performed in the kidney parafilm sections of control and diabetic CD-1 and C57Bl6 mice. Scale bar 50 µM. Representative pictures are shown here. N=6 were evaluated for in each group. **h.** Blood glucose level was evaluated in the scramble and sirt3 siRNA transfected diabetic mice before the sacrifice of mice. **i.** Body weight. **j.** Kidney weight (mg/g body weight). **k.** Albumin creatinine ratio. **l.** Cystatin C (pg/ml). N=6 were analyzed in each group. **m.** Immunostaining of smad3 phosphorylation, α-SMA and TGFβR1 in the scramble and sirt3 siRNA transfected diabetic CD-1 mice. Representative pictures are shown here. Scale bar 50 µM. N=6 were analyzed. **n.** Western blot analysis of PGC1α and CPT1A in the control and diabetic group of CD-1 and C57Bl6 mice. Representative blots from four blots have been shown here. Densitometry calculations were analyzed by ImageJ program. N=6 were analyzed in each group. Data in each graph are shown as mean±SEM. Tukey test was performed to analyze the statistical significance.

**Supplementary Figure 2 SIRT1 knockdown in diabetic CD-1 mice**

**a**. Body weight, blood glucose level, kidney weight, albumin to creatinine ratio and cystatin C level in the scramble siRNA and sirt1 siRNA injected CD-1 mice. **b**. Immuhistochemical staining of SIRT1 protein in the parafilm section of kidneys of scramble and sirt1 siRNA injected mice. Representative pictures are shown here. N=5 were analyzed in each group. **c**. Western blot analysis of SIRT1 and α-SMA protein level in the kidneys of scramble and sirt1 siRNA injected mice. Representative from 4 blots is shown here. Densitometric calculations were normalized by β-actin. **d-e**. Masson trichrome and Sirius red staining in the parafilm section of kidneys of scramble and sirt1 siRNA injected mice. Relative area fibrosis and relative collagen deposition were calculated by image J program. N=6 were analyzed in each group. **f**. Immuno-staining of α-SMA, smad3 phosphorylation and TGFβR1 proteins were analyzed in the section of kidneys of scramble and sirt1 siRNA injected mice. N=5 were analyzed in each group. Data in each graph are shown as mean±SEM. Tukey test was performed to analyze the statistical significance.

**Supplementary Figure 3 Sub-cellular distribution of SIRT1 and SIRT3 in the kidneys of control and diabetic mice**

**a**. Schematic chart of sub-cellular fractionation. **b**. Western blot analysis of SIRT1 and SIRT3 protein level in the nuclear, cytosolic and mitochondrial fractions of kidneys of control and diabetic mice. Representative from 3 blots is shown here. N=3 were analyzed.

**Supplementary Figure 4 Fibrogenic phenotype is associated with higher level of abnormal glycolysis**

**a**. Immuno-staining of GLUT1, HK2, PKM1, PKM2, PDK4 in the control and diabetic mice of CD-1 and C57Bl7Bl6 strains. **b**. Immuno-staining of GlUT1, HK2, PKM2 and PDK4 in the parafilm section of kidneys of scramble siRNA and sirt1 siRNA injected mice. N=5 were evaluated in each group. Representative picture is shown here. Scale Bar 50 µM.

**Supplementary Figure 5** **Abnormal glycolysis is associated with mesenchymal transformations**

**(a)** Gene expression analysis of TGFβ1 and snail1 in the kidneys of control and diabetic mice of CD-1 and C57Bl6 mice strain. Control n=5, DM n=6 were analyzed. **(b)** Co-immunolabelling of HK1, PKM1, and PDK1 with α-SMA, were analyzed by using fluorescence microscope in the kidney of control and diabetic mice of CD-1 and C57Bl6 mice. N=3 were analyzed in the case of control while N=5 were analyzed in the diabetic group of both strains. Representative figure in each panel has been shown. **(c)** Gene expression analysis of TGFβ1 and snail1 in the kidneys of scramble and sirt3 siRNA injected kidney of diabetic CD-1. Scramble n=4, Sirt3 siRNA n=5 were analyzed. Data in the graph are expressed as the mean±SEM. Tukey test was performed to calculate statistical significance.

**Supplementary Figure 6 Schematic diagram indicating the action of glycolysis inhibitors (DCA and 2-DG)**

**Supplementary Figure 7 Physiological characteristics of glycolytic inhibitors (DCA and 2-DG) treatment in mice**

**a-b**. On Blood glucose. **c-d**. Blood pressure. **e-f**. Body weight. **g-h**. Kidney weight. **i-j**. Albumin to creatinine ratio **k-l**. Cystatin C. **m.** Densitometry calculation of CollaA, α-SMA, Vimentin, and PGC1α in the kidney of DCA and 2-DG treated diabetic mice. The values were normalized by β-actin. **n.** Densitometry calculation of key glycolysis enzymes HK2, PKM2, PDK4 and SIRT3 protein level in the kidney of DCA and 2-DG treated diabetic mice. The values were normalized by β-actin. Data in each graph are shown as mean±SEM. Tukey test was performed for calculation of statistical significance.

**Supplementary Figure 8 DCA and 2-DG treatment in the control and diabetic C57Bl6 mice**

**a**. Change in body weight, blood glucose, kidney weight and MTS were analyzed in the DCA and 2-DG treated-diabetic C57Bl6 mice. Representative image is shown here. N=5 were analyzed in each group. **b**. Western blot analysis of SIRT1, SIRT3 protein level in the kidneys after DCA and 2-DG treatment in the diabetic C57Bl6 mice. Representative from four blots is shown here. N=5 were analyzed in each group. **c**. Immunostaining of SIRT1 protein in the parafilm section of kidneys of control, diabetes, DCA-treated diabetic group and 2-DG-treated-diabetic group in the CD-1 mice. N=5 were analyzed in each group. Data in each graph are shown as mean±SEM. Tukey test was performed for calculation of statistical significance.

**Supplementary Figure 9 Glycolysis inhibition in TGFβ1 induced HK-2 cells**

**a.** Densitometry analysis of FSP-1, α-SMA, TGFβR1 and smad3 phosphorylation in the DCA treated cells stimulated with or without TGFβ1. Three independent set of the experiment were analyzed. **b-c.** Densitometry analysis of GLUT1, HK2, PKM2, PDK4, SIRT3 and PGC1α in the DCA treated cells stimulated with or without TGFβ1. Three independent set of the experiment were analyzed. **d.** Western blot analysis of CPT1A. Four independent sets of experiment were performed. **e.** Densitometry calculation of the α-SMA, TGFβR1, SIRT3, HK2, PKM2 and HIF1α protein level in the HK2 siRNAs, PKM2 siRNAs and HIF1α siRNAs transfected HK-2 cells. Densitometry calculations were calculated from five blots by ImageJ program. Three independent experiments were performed. **f.** Densitometry calculation of the SIRT3, FSP-1, α-SMA, smad3 phosphorylation, TGFβR1, GLUT1, HK2, PKM2 and PDK4 protein level in the scramble siRNAs and sirt3 siRNAs transfected HK-2 cells. Densitometry calculations were calculated from four blots by ImageJ program. Three independent experiments were performed. **g.** Densitometry calculations of α-SMA, HK2, PKM2, PDK4 and HIF1α in the DCA treated scramble and sirt3 siRNA transfected HK-2 cells. Densitometry calculations were calculated from three blots by ImageJ program. Three independent experiments were performed. Data in the graph are represented as mean±SEM. Tukey test was performed for calculation of statistical significance.

**Supplementary Figure 10 SIRT3 deficiency is linked with higher level of VEGF protein level in the diabetes-associated kidney fibrosis**

**a**. Western blot analysis of VEGF protein in the kidney of control and diabetic CD-1 mice. Representative from 3 blots are shown here. **b**. Western blot analysis of VEGF protein in the kidney of scramble siRNA and sirt3 siRNA injected mice. Representative from 3 blots are shown here. Data in the graph are represented as mean±SEM. Tukey test was performed for calculation of statistical significance.
